# Photomodulation Approaches to Overcome Antimicrobial Resistance

**DOI:** 10.3390/ph16050682

**Published:** 2023-05-02

**Authors:** Sofia N. Sarabando, Andreia Palmeira, Maria Emília Sousa, Maria Amparo F. Faustino, Carlos J. P. Monteiro

**Affiliations:** 1 Laboratory of Organic and Pharmaceutical Chemistry, Chemical Sciences Department, Faculty of Pharmacy, University of Porto, 4050-313 Porto, Portugal; sofia.sarabando@ua.pt (S.N.S.); apalmeira@ff.up.pt (A.P.); 2 LAQV-Requimte and Department of Chemistry, University of Aveiro, 3010-193 Aveiro, Portugal; faustino@ua.pt; 3 CIIMAR—Interdisciplinary Centre of Marine and Environmental Research, 4450-208 Porto, Portugal

**Keywords:** photopharmacology, photoswitch, photocaged, porphyrin, antimicrobials

## Abstract

Photopharmacology is an approach that aims to be an alternative to classical chemotherapy. Herein, the different classes of photoswitches and photocleavage compounds and their biological applications are described. Proteolysis targeting chimeras (PROTACs) containing azobenzene moieties (PHOTACs) and photocleavable protecting groups (photocaged PROTACs) are also mentioned. Furthermore, porphyrins are referenced as successful photoactive compounds in a clinical context, such as in the photodynamic therapy of tumours as well as preventing antimicrobial resistance, namely in bacteria. Porphyrins combining photoswitches and photocleavage systems are highlighted, taking advantage of both photopharmacology and photodynamic action. Finally, porphyrins with antibacterial activity are described, taking advantage of the synergistic effect of photodynamic treatment and antibiotic therapy to overcome bacterial resistance.

## 1. Introduction

**Figure 1:**
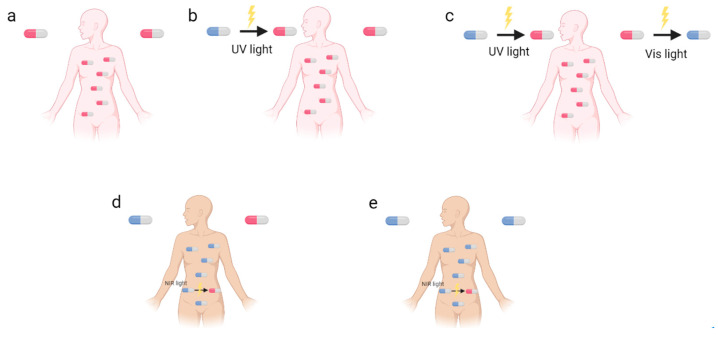
Comparison of the principle behind (**a**) classical chemotherapy, (**b**) photopharmacology using UV light—PPG, (**c**) photopharmacology using UV and visible light—photoswitches, (**d**) photopharmacology using IR light—PPG, and (**e**) high-precision photopharmacological chemotherapy using IR-light—photoswitches. Red capsule denotes cytotoxic effects and blue capsule is an inactive form and not cytotoxic.

Photopharmacology is a new pharmacophore modality that is gaining attention as a promising alternative to classical chemotherapy drugs (Figure 1a) [1,2,3]. It is characterized by the combination of photochemistry and pharmacology, and it aims at solving issues related to poor drug selectivity, minimizing off-target effects and the emergence of drug resistance [4]. With the increased rate of antimicrobial resistance, there are fewer treatments available, so the World Health Organization (WHO) recommends developing new antimicrobial approaches to overcome this problem [5]. In this way, the use of light on the inactivation of microorganisms (MO) has been shown to be an outstanding tool, as it is non-evasive and capable of fast and controllable delivery to precise locations, paving the way for the use of photons for new medical treatment approaches, such as photopharmacology, an emerging field that utilizes light to control biological processes with high spatiotemporal precision, offering a promising strategy for overcoming antimicrobial resistance [2,6]. Its principle is the introduction of a photocaged or photoswitchable moiety into the molecular structure of the bioactive compound, where the light effect can be irreversible or reversible. Photocleavage molecules are an example of irreversible photoactivation by using photocleavable protecting groups (PPG) as ‘’cages’’ [7]. PPG are small moieties that can be released upon irradiation, exposing the bioactive molecule in its active form [7]. On the other hand, photoswitches are molecules that can be interconverted upon the action of light, in a reversible and fast process, into different structural configurations (*cis* or *trans*), in which one of the configurations is the bioactive form [8]. Light offers the possibility to control both the pharmacokinetic (PK) and pharmacodynamic (PD) properties of molecules, switching the drug from a low to high affinity with the biological target (*cis* and *trans*, in case of photoswitches) and allowing molecular activation in case of PPG. Therefore, light can improve selectivity, being particularly suitable when the disease is localized, and consequently reduces adverse effects by adjusting its wavelength and intensity [4,9,10]. One of the major challenges in photopharmacology, using short-wavelength light (ultraviolet (UV), below 350 nm), is the limited penetration into tissue and off-target toxicity due to the fact that UV light is toxic to healthy cells (Figure 1b,c). At a first glance, utilizing UV light may not appear to be a favorable strategy. Nevertheless, the primary advantage of this approach is the mitigation of cytotoxicity once the photoswitches lose their activity after the therapeutic effect (Figure 1c). A high-precision photopharmacological approach utilizing long-wavelength light (near infrared, 650–900 nm) can effectively overcome the UV light problem. This approach has the added advantage of deeper tissue penetration without harming healthy cells, as it specifically targets only the desired treatment area (Figure 1d,e). Photoswitches, due to their reversible process, can also minimize and prevent side effects, such as generalized cytotoxicity and antimicrobial resistance (Figure 1e).

Photodynamic therapy (PDT) is a spatiotemporal therapeutic modality approved by the Food and Drug Administration (USA-FDA) and European Medicines Agency (EMA) to treat skin diseases and other organs where light can reach [1,11,12,13,14,15,16]. It requires the administration of a photosensitizer (PS), which is a non-toxic drug that is accumulated in the target cells and activated by adequate visible light irradiation in presence of dioxygen (O_2_) to produce reactive oxygen species (ROS) that cause cytotoxic effects at the therapeutic target. Briefly, the mechanism of action of PDT is based on PS promotion by light absorption from the ground state (^1^PS) to an excited state (^1^PS*). ^1^PS* can return to the ground state (^1^PS) directly through the emission of light (fluorescence) or heat (internal conversation), or indirectly by intersystem crossing, converting to the excited triplet state (^3^PS*). In this state, ^3^PS* can interact with O_2_ by two different mechanisms. The type I mechanism involves electron transfer to lead to the production of free radicals, which interact with O_2_ and produce ROS, such as hydrogen peroxide (H_2_O_2_), hydroxyl radicals (HO^•^), and superoxide radicals (O_2_^•−^). The type II mechanism involves energy transfer between the PS excited and the ground state dioxygen (^3^O_2_), producing singlet oxygen (^1^O_2_). As ^1^O_2_ is an uncharged molecule, it can diffuse through plasma and biological membranes and will trigger a chain of successive oxidations of biological molecules (e.g., DNA, lipids, and proteins) [17,18,19,20,21]. It is believed that the production of ROS through the type II reaction is predominant in PDT. This photodynamic approach was also used with great success on the photoinactivation of microorganisms and demonstrates great advantages when compared with the conventional antimicrobial agents, namely (i) the non-induction of resistance mechanisms of the microbial cells and (ii) either resistant and sensitive strains are responsive to the photodynamic treatment and (iii) can be applied to inactivate a range of microbial entities (bacteria, fungi, viruses, protozoa, parasites [11,12,22,23,24,25,26,27,28,29], among others). The versatility of PDT is not limited to clinical applications [23,30]. This technique is valuable for clinical [31,32,33] and non-clinical applications, such as removing biofilms from medical devices and implants [34] and environmental water treatments [35,36,37].

Herein, different classes of photoswitches and photocleavage compounds and their biological applications will be described. Furthermore, porphyrins will be referred to as successful photoactive compounds for use in antimicrobial photodynamic treatment (aPDT), as well as promising approaches when combined with photoswitches or photocleavage to take advantage of both photopharmacology and PDT. Finally, examples combining aPDT and antibiotic therapy will be reported, highlighting this application to overcome resistance.

## 2. Different Classes of Photoswitches and Some Examples in Antibacterial Applications

Photoswitches are compounds that can be interconverted into two different structural configuration forms by using light. These compounds are of great importance in several fields, including molecular electronics, phytopharmacology, and catalysis [38]. In 1937, Hartley discovered that *trans* azobenzene could come from the *cis* isomer by UV-light and, conversely, by keeping the molecule in the dark, or irradiating it with blue light, the thermodynamically more stable *trans* isomer could be recovered [39]. The *trans* → *cis* photoisomerization of azobenzenes can occur by n → π* excitation (lower energy) at 380–500 nm, or *π* → π* excitation (higher energy) at 280–380 nm [40].

By coupling a photoswitch moiety with a biologically active compound, it is possible to turn ON (high-affinity form) and OFF (low-affinity form) this bioactive compound. In fact, azobenzene-based photoswitches are the most widely used due to their efficacy, reversibility, and repeatability. However, photoswitchable molecules show incomplete photoisomerization, insufficient light sensitivity for some applications, and difficulties in fine-tuning to produce a maximal difference between ON and OFF states of the compounds [41].

**Figure 2:**
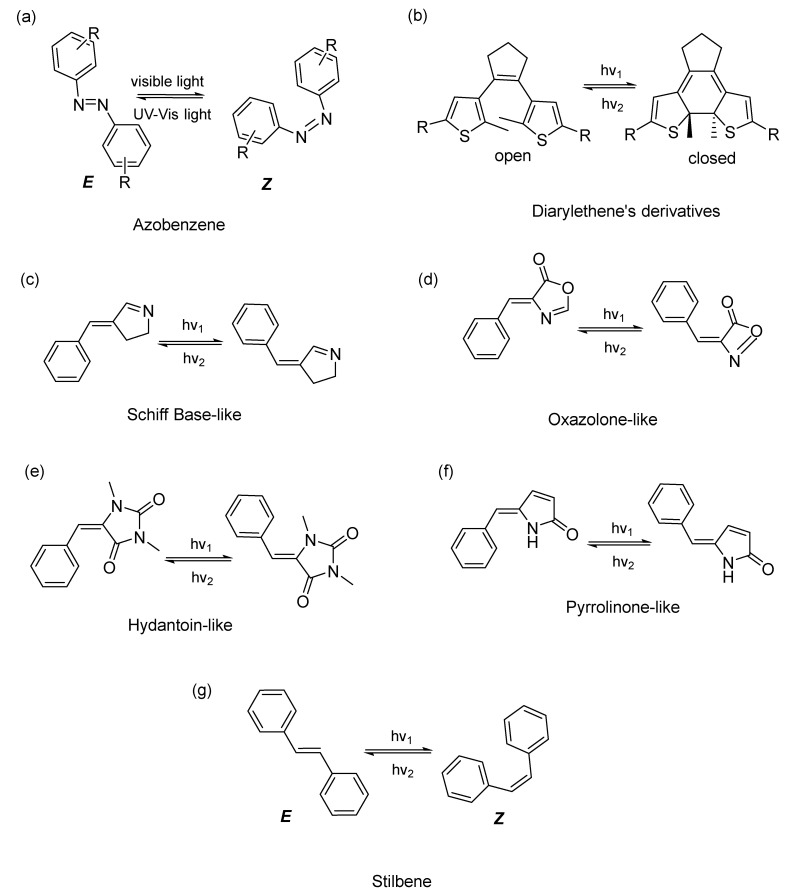
Structures of some photoswitches and their photoisomerization process. h*ν* is the photon energy (h—Planck’s constant; *ν*—frequency; *ν*_1_ *< ν*_2_).

Ideal photoswitches are molecules that absorb visible light and have a long half-life time of the metastable isomers [42]. Although UV-light is typically used, studies have shown that azoarenes can be activated by visible light [42] and near infrared (NIR) light, which is required to penetrate tissues [39]. Diarylethenes [4], Schiff base-like [43], oxazolone-like [44], hydantoin-like [45], pyrrolinone-like [46], and stilbene [47] compounds are also types of photochromic compounds (Figure 2).

### 2.1. Azobenzenes

**Figure 3:**
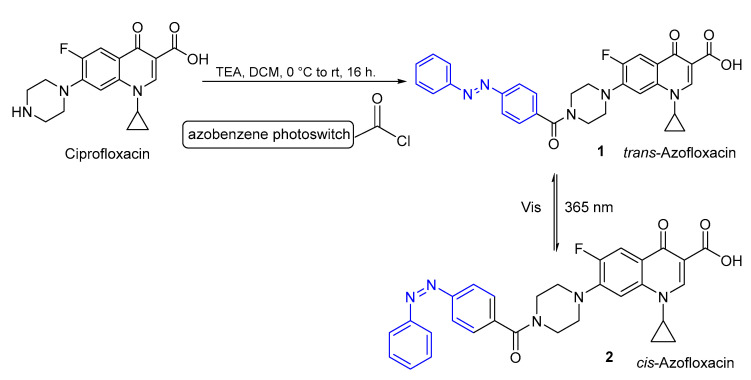
Molecular structure of ciprofloxacin and its photoswitchable analogue, azofloxacin.

Azobenzenes are a class of compounds that present an aromatic molecular structure, characterized by the presence of sensitivity to external stimuli -N=N- double bond, with the appropriate features for incorporation into most pharmacophores [48,49]. Feringa et al. [9] investigated the possibility of obtaining pharmacological agents by conjugating photoswitches with ciprofloxacin (Figure 3) using azobenzenes to treat local diseases, such as a solid tumor or local inflammation. Structure activity relationship (SAR) studies indicated that the secondary amine in the piperazine ring of ciprofloxacin can be modified without loss of activity; therefore, this position was chosen for conjugation with the azobenzene moiety (Figure 3). This approach allowed for the preparation of an antibiotic with photoswitchable activity (azofloxacin). Before irradiation, azofloxacin occurs in 100% as the *trans*-isomer (compound **1**), but after irradiation with light at 365 nm, 61% of azofloxacin converts to the *cis-*isomer (compound **2**). The non-total conversion of *trans* azofloxacin to *cis* azofloxacin might be due to the aggregation between azobenzene and molecules in an aqueous environment, not allowing complete photoisomerization [9].

The bacterial activity of azofloxacin was assessed on *Escherichia coli (E. coli)* CS1562 and *Micrococcus luteus* (*M. luteus*) ATCC 9341, before and after irradiation with light at 365 nm. Ciprofloxacin was chosen as the control. The azofloxacin analogue proved to be more efficient than ciprofloxacin in *M. luteus* (Table 1), specially before irradiation (*trans*-azofloxacin, compound **1**), which can be modulated by exposure to light, giving rise to *cis*-azofloxacin (compound **2**). While no significant changes were observed on the Gram-negative bacterial model *E. coli*, the antibacterial activity of *trans*-azofloxacin on the Gram-positive bacterial model *M. luteus* was found to be about 50 times greater than the native drug ciprofloxacin.

The introduction of the azobenzene moiety into an antibacterial drug allows the light to spatiotemporally control the antibacterial activity and presents plenty of opportunities to explore new targets in photopharmacology. According to the authors hypothesis, although on *E. coli* the results are not promising, it is still an alternative to treatment, even when used in combination with chemotherapy. Clearly, further studies would be needed to confirm this hypothesis.

**Figure 4:**
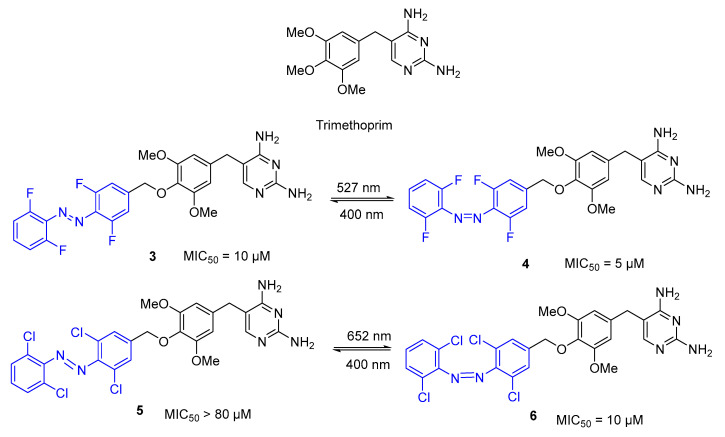
Red-shifted responsive trimethoprim derivatives and respective MIC_50_ values.

Wegener et al. [50] reported for the first time a red-shifted responsive azobenzene photoswitch containing the antibiotic trimethoprim as a core (Figure 4). Trimethoprim interferes with the biosynthesis of folate by inhibition of dihydrofolate reductase (DHFR), which catalyses the reduction of dihydrofolate to the active cofactor tetrahydrofolate [51,52]. Therefore, the biosynthesis of the amino acids glycine and methionine, as well as purines and thymidine triphosphate are impaired. Of note, trimethoprim has a higher selectivity to bacterial DHRF than mammalian DHFR and it is active against both Gram-positive and Gram-negative bacteria to treat urinary and respiratory tract infections. Nevertheless, with the emergence of bacterial resistance, it is important to develop new therapeutic entities, such as photoresponsive analogues, a strategy that can be undertaken by modifying the structure of trimethoprim without reducing bacterial DHFR affinity. Based on the aforementioned results, the authors prepared a red-shifted analogue (azobenzene substituted with fluorine or chlorine at ortho-positions), where the photoisomerization with visible light was possible, while maintaining the same antibacterial properties (Figure 4). These substituents allowed the effective use of lower-energy n → π* excitation to trigger *trans* → *cis* photoisomerization. The photostationary state (PSS) of tetra-fluoro-substituted azobenzene was found to have an 89:11 ratio of *cis:trans* at 527 nm (visible light). In the case of tetra-*o*-chloroazobenzene, irradiation with 652 nm (near-infrared light) produced photoisomerization to a PSS of 87:13 proportion of *cis:trans.*

The effectiveness of antibacterial activity against *E. coli* was evaluated before and after irradiation. Even though compounds **5** and **6**, which contain trimethoprim tetra-*o*-chloroazobenzene substitution, exhibited less photoisomerization than compounds **3** and **4**, which contain tetra-*o*-fluoro-substituted azobenzene, they were still of interest. Compound **5** displayed no activity with MIC_50_ > 80 μM, nevertheless it was responsive to irradiation (652 nm), giving compound **6**, which induced bacteriostasis with MIC_50_ = 10 μM. 

However, tetra-*o*-fluoro-substituted azobenzene is better at inducing bacteriostasis with an MIC_50_ = 5 μM. Furthermore, it was important to study the photoswitching reversibility to the *trans* isomer (inactive) found when blue light at 400 nm was used.

**Figure 5:**
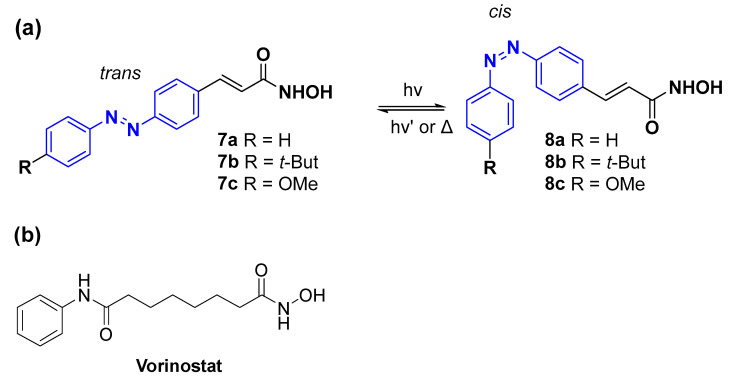
Figure **5.** (**a**) Structures of *trans* and *cis* azobenzene HDAH inhibitors and (**b**) structure of Vorinostat, inhibitor of histone deacetylation, 2 h half-life.

Amidohydrolase enzymes are virulence factors found in various pathogenic bacteria responsible for a great number of hospital-acquired infections and deaths. Weston and co-workers [53] reported inhibitors of these enzymes using azobenzenes as photoswitches (Figure 5). Amidohydrolase enzymes are homologous to the human histone deacetylases (HDACs); therefore, with the knowledge of the pharmacophore of HDAC inhibitors [54,55,56], the crystal structure of the enzyme active site of the HDAC family [57,58,59], and knowing the bacterial enzymes B/A-HDAH (histone deacetylase-like amidohydrolase from Bordetella/Alcaligenes [59]), APAHs (acetylpolyamine amidohydrolases from *Pseudomonas aeruginosa* (*P. aeruginosa*), PA1409 and PA0321 [58]), and PA-HDAHs (acetylpolyamine amidohydrolases from *P. aeruginosa*, PA3774), the authors designed and conjugated a pharmacophoric moiety with a zinc chelating group (hydroxamic acid), a phenyl capping unit, and an azobenzene linker.

With the photoisomerization of the azobenzene compounds **8** activated by a UV-A lamp at 365 nm, the half-maximal inhibitory concentration values (IC_50_) against the bacterial enzymes B/A-HDAH, PA-HDAH, and HPAHs were assessed using SAHA (suberoylanilide hydroxamic acid, also known as vorinostat, Figure 5b) as a positive control (Table 2). It should be noted that incomplete photoswitching is an issue, and about >10% of the former isomer was present upon irradiation.

In general, compounds **7a** and **7c** showed better activity than the positive control against B/A-HDAH. However, treatment with UV-A light was not successful as the cis-isomer was more effective than the *trans*-isomer. Compound **8a** revealed a better efficacy than the positive control against the PA-HDAH and APAHs. Here, it was demonstrated that the efficacy of the cis-isomer obtained after UV-A light treatment is greater than the trans-isomer. Compound **7b** exhibits a greater steric effect due to the presence of the *tert*-butyl group, which leads to an unfavourable steric fit. As a consequence, its inhibitory activity is weaker than that of compounds **7a** and **7c**.

Initially, the authors proposed that the *cis*-isomers would induce more unfavourable steric interactions, reducing inhibitory activity, so the *trans*-isomer would be the most active. However, this was not observed, and the results showed that the most active isomer depends on the inhibitor substituent and the enzyme (the *cis*-isomers were found to be better in APAHs).

**Figure 6:**
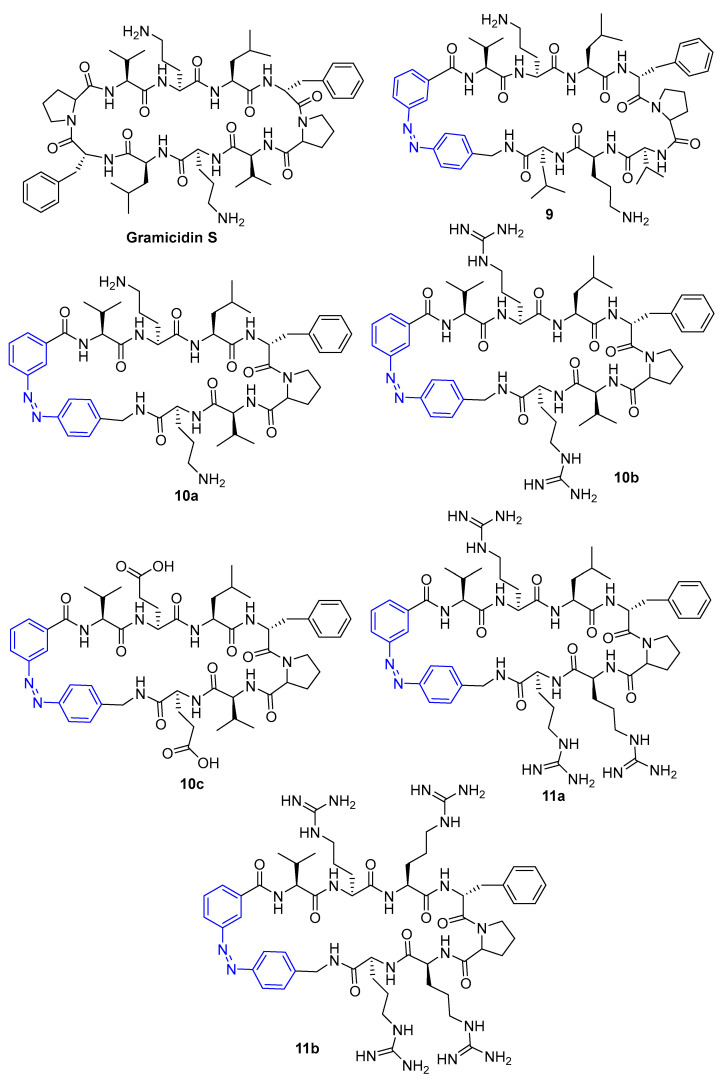
Structure of Gramicidin S and its modified peptides (compounds **9**–**11**) containing azobenzene units [61].

Gramicidin S is a cyclic peptide known for its antibacterial activity [60]; therefore, Yuan et al. [61] developed an approach based on an azobenzene moiety containing gramicidin S to modulate, reversibly, the antibacterial activity (Figure 6).

Peptide **9** was designed by replacing proline and D-phenylalanine amino acid residues with an azobenzene moiety, maintaining the amphiphilic side chain for biological activity. Peptide **10a** was obtained by removing the leucine residue in peptide **9**. Arginine (basic amino acid residue) and glutamic acid (acidic amino acid residue) were replaced in peptide **10a**, to produce peptides **10b** and **10c**, respectively, aiming to investigate possible differences in the antibacterial activity due to the positively or negatively charged amino acid residues. To study the amphiphilicity on the antibacterial activity, basic amino acid residues were incorporated into the peptide backbone by replacing arginine with leucine and valine residues (peptides **11a** and **11b**). When cis-isomers of peptides were irradiated with blue light (405 nm), *trans*-isomers are obtained. On the other hand, the *trans*-enriched PSS of **9–11** were irradiated by UV-A light of 352 nm for 2 h to convert to the respective *cis*-enriched PSS.

The MIC values of gramicidin S and its peptides were determined against *Staphylococcus aureus (S. aureus)* ATCC 49775 (Table 3).

Peptide **9** only has two intramolecular strong hydrogen bonds linking the upper and lower strand, showing a distorted β-strand secondary structure, proved in a molecular modelling study. Gramicidin S has four strong hydrogen bonds, while the *cis*-isomers of peptides **10** and **11** have three strong hydrogen bonds, causing a well-defined secondary structure. The *trans*-isomers of peptides **10** and **11** lack intramolecular hydrogen bonding and, therefore, show a weak secondary structure. The *cis*-isomers of peptides **10a** and **10b** proved to be more efficient than *trans*-isomers, with *cis*-enriched **10b** exhibiting the highest antibacterial potential in this study. Similar to peptide **10c**, peptides **11a** and **11b** were inactive, which was attributed to the replacement of hydrophobic residues (valine and leucine), which may disrupt the amphiphilic nature of peptides, decreasing penetration through the bacterial membrane. In this study, the use of light was not essential to activate the ON effect, since the *trans*-isomer was not more efficient than the *cis*-isomer. However, light would be a good strategy to generate an OFF effect and, therefore, reduce side effects.

### 2.2. Diarylethenes

**Figure 7:**
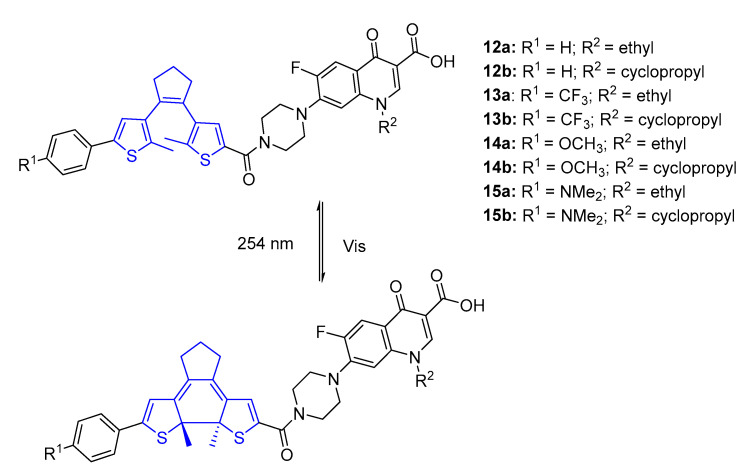
Diarylethenes based on fluoroquinolone antibiotics [64].

Similar to azobenzenes, diarylethenes have an aromatic molecular structure that allows perfect incorporation into most pharmacophores. Thus, diarylethenes have been studied and applied as photoswitch molecules that can be interconverted by modulation between open and closed forms [62,63]. Li et al. [64] developed an approach of molecular hybridization by introducing a diarylethene moiety into fluoroquinolone (norfloxacin, R^2^ = ethyl and ciprofloxacin, R^2^ = cyclopropyl) to obtain switchable antibacterial agents (Figure 7). As mentioned before, SAR studies showed that the secondary amine in the piperazine ring of fluoroquinolones can be modified without loss of antibacterial activity, so this position was chosen for conjugation with a diarylethene moiety.

The antibacterial activity of the diarylethene derivative was assessed before and after irradiation with UV-C light (254 nm) by performing MIC values on *E. coli* and *S. aureus* (Table 4).

All the open-isomers showed a lower antibacterial activity on *E. coli* (MIC = 16–32 μg mL^−1^) than closed-isomers. A similar activity against *S. aureus* (MIC = 8–32 μg mL^−1^) is shown in both the open and closed forms. However, all of the compounds were less active than the native drugs, norfloxacin and ciprofloxacin (MIC = 0.125 μg mL^−1^). The activity decrease can be related to the addition of the switchable moiety. Particularly, compounds **12b** and **14b** showed a great difference in the antibacterial activity against *E. coli* before and after irradiation, with the closed form being 16-fold more efficient than the open form. The ring-open form in diarylethenes shows a flexible conformation due to free rotation around the C–C bonds joining the thiophene to the central cyclopentene ring, consequently adopting various geometries. This flexible conformation was hypothesized not to allow the formation of stable complexes with DNA gyrase, decreasing the open-isomer antibacterial activity. Furthermore, the closed-form of compounds **12b** and **14b** appear to have a rigid conformation, which allows the formation of stable complexes with the DNA gyrase of *E. coli* and, consequently, derivatives were shown to increase the antibacterial activity up to 16-fold. Despite **12b** and **14b** being less effective than the parent drug ciprofloxacin, they showed the potential to be used as novel switchable antibacterial agents [64].

### 2.3. PHOTACs

**Figure 8:**
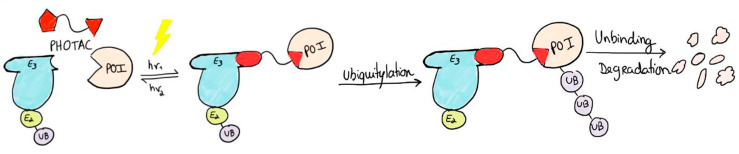
Schematic of the working model of a PHOTAC. The molecules toggle between an inactive form and an active form upon irradiation. Red pentagon does not fit in E3 ubiquitin ligase complex until irradiation occurs. There, the conformation changes, the ligand fits, and the ubiquitylation process occurs. Adapted with permission from [66]. Copyright, 2020, Elsevier.

PROTACs (**PRO**teolysis **TA**rgeting **C**himeras) are bifunctional small molecules that have been extensively studied as a novel method to treat diseases that result from the aberrant expression of specific disease-associated proteins [65]. PROTACs can degrade these proteins selectively by ubiquitination. This approach is promising for the treatment of cancer, neurodegenerative diseases, inflammatory diseases, and viral infections. By the combination of PROTACs with photopharmacology, a new approach arises—**PH**otoswitchable proteolysis **TA**rgeting **C**himeras (PHOTACs), which are bifunctional small molecules that target a protein of interest (POI) for ubiquitylation by an E3 ubiquitin ligase complex, promoting proteasome degradation (Figure 8). This approach allows the change in the reversibly endogenous protein levels, being helpful for potential treatments of diseases, such as cancer [66] and eventually Alzheimer’s disease [67]. Several studies [68,69,70] have already designed and synthesized PHOTACs by incorporating photoswitches, such as azobenzenes, into PROTACs.

**Figure 9:**
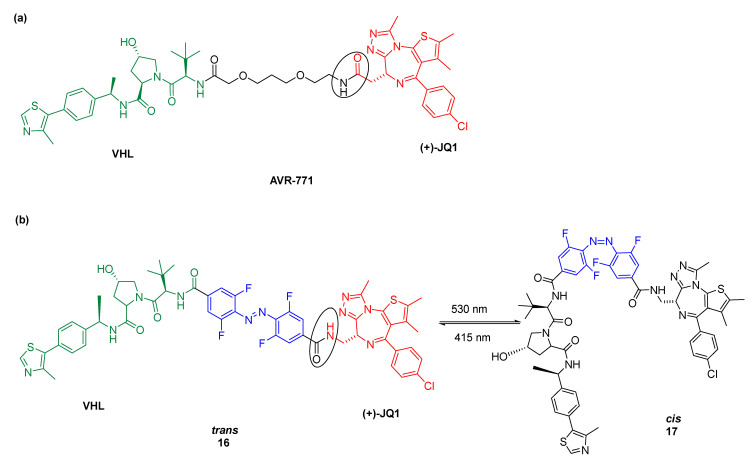
Figure **9.** (**a**) structure of PROTAC AVR-771 and (**b**) schematic representation of photoisomerization between *trans*-isomer (active, compound **16**) and *cis*-isomer (inactive, compound **17**) [70].

**Figure 10:**
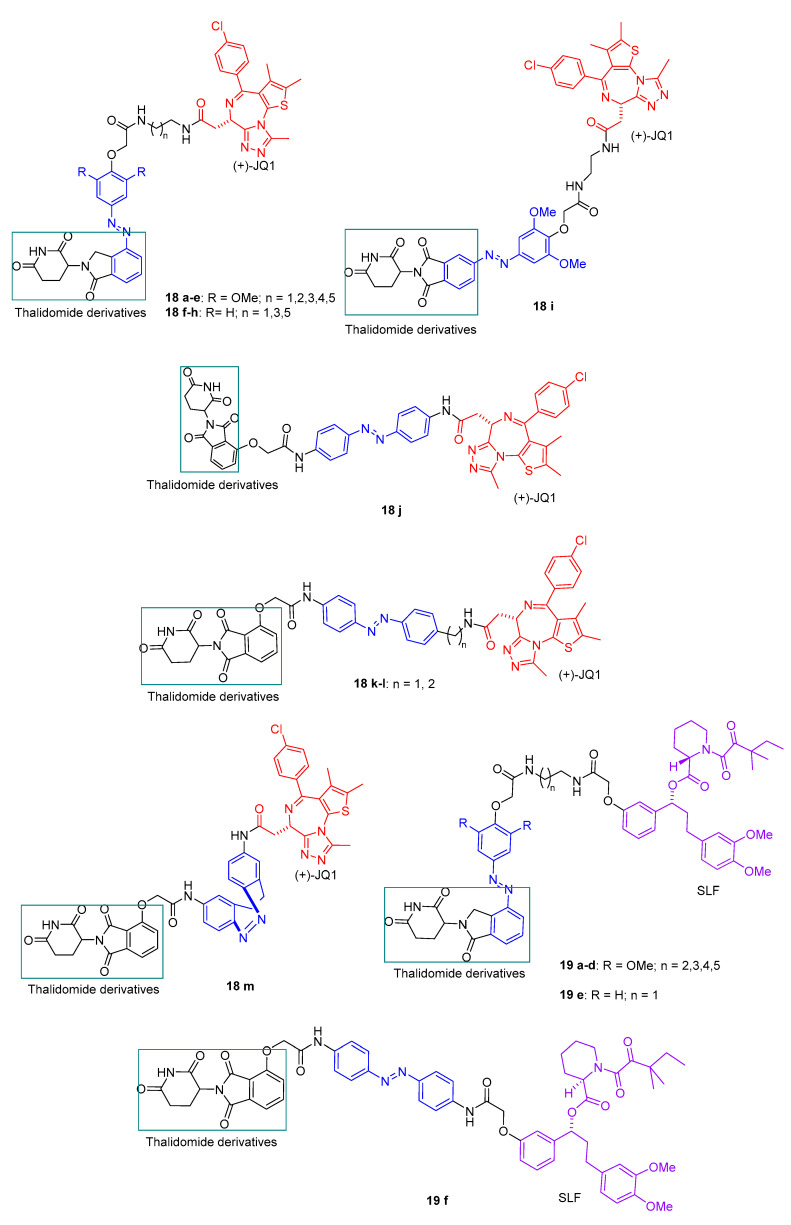
Structures of PHOTACs **18** and **19** series.

Pfaff et al. [70] were the first to report a novel approach based on PROTACs, including *o*-F_4_-azobenzene linkers. AVR-771 is a PROTAC that degrades BRD2 and BRD4 (BET proteins), so it has (+)-JQ1 as a POI ligand and VHL (Von Hippel-Lindau) as a ligand of E3 ubiquitin ligase (Figure 9a). Based on AVR-771, a novel PHOTAC was designed (Figure 9b). The biological activity of PHOTACs was tested in Ramos cells and proved to be promising. While the *trans*-isomer was active in decreasing BRD2 levels, the *cis*-isomer did not induce protein degradation, hypothesized due to the shorter distance between BRD2 and VHL ligands because of the ‘’compact form’’ of the *cis*-isomer (Figure 10). Despite the ARV-771 PROTAC being able to degrade both BRD2 and BRD4, degradation of BRD4 was not observed for photoPROTAC. This is hypothesized due to the reversed amide bond between (+)-JQ-1 and the *o*-F_4_-azobenzene moiety. 

Reynders et al. [68] also reported the photopharmacological application to target protein degradation by incorporating azobenzenes into PROTACs, promoting light-dependent proteolysis. In the dark, the molecules do not have proteolytic activity, but upon irradiation with blue-violet light (380–440 nm), they were found to be activated. The azobenzene moiety was introduced on the phthalimide-conjugated ligand to bind cereblon E3 ubiquitin ligase complex to allow the affinity change. Based on the previously reported PROTAC dBET1 (Figure 10a) [71], a POI ligand, (+)-JQ1, was chosen for the design of the compound **18** series. (+)-JQ1 is an inhibitor of BET proteins BRD2-4. For the design of the compound **19** series, a synthetic ligand of FK506-binding protein (SLF) was chosen as the POI ligand based on PROTAC dFKBP-1 (Figure 10b), reported previously. The SLF ligand is a synthetic ligand for FKBP inhibitors. Figure 10 summarizes the small library of PHOTACs synthesized in this work.

**Figure 11:**
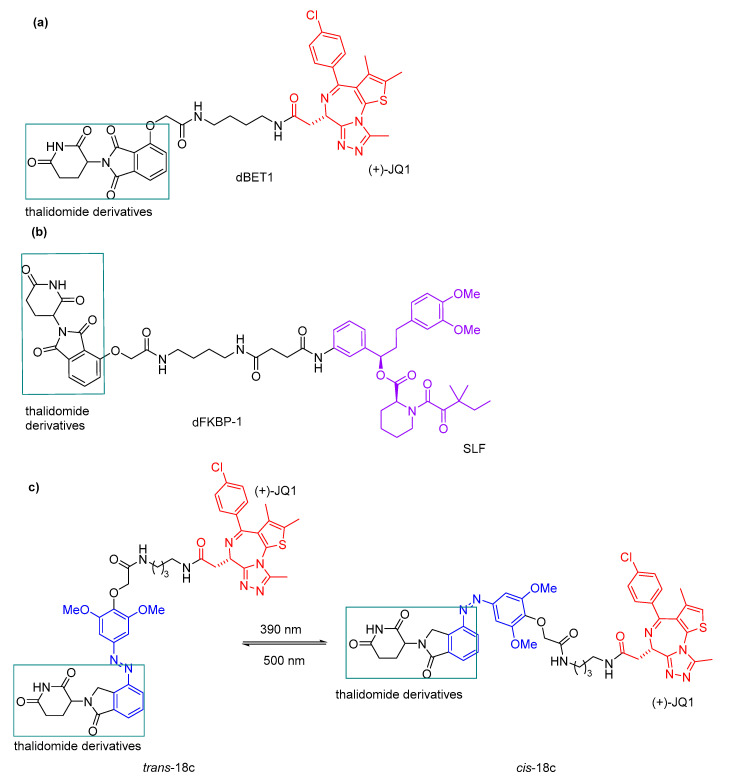
Structure of PROTACs (**a**) bBET1 and (**b**) dFKBP-1. (**c**) Photoswitching of compound **18c**.

The compound **18** series was evaluated on the viability of RS4;11 lymphoblast leukemic cells, in which compound **18c** (Figure 11c), with a 1,4diaminobuthyl spacer, proved to be the most effective. Therefore, BET protein (BRD2-4) levels were analysed in the presence of compound **18c**. A decrease in BRD4, BRD3, and BRD2 levels upon compound **18c** irradiation (390 nm) was observed, but not in the dark. A light dependent BRD4 degradation was also demonstrated in breast cancer cell lines (MBMDA231 and MBMDA468) using compound **18c**. Upon irradiation with UV-A light (390 nm), *trans*-**18c** was triggered to *cis*, obtaining a PSS of >90%. When the *cis*-isomer was irradiated with green light (500 nm), a PSS of >70% *trans* was obtained (Figure 11c). The compound **19** series was tested on FKBP12 levels; however, a slight activity in the dark was observed.

**Figure 12:**
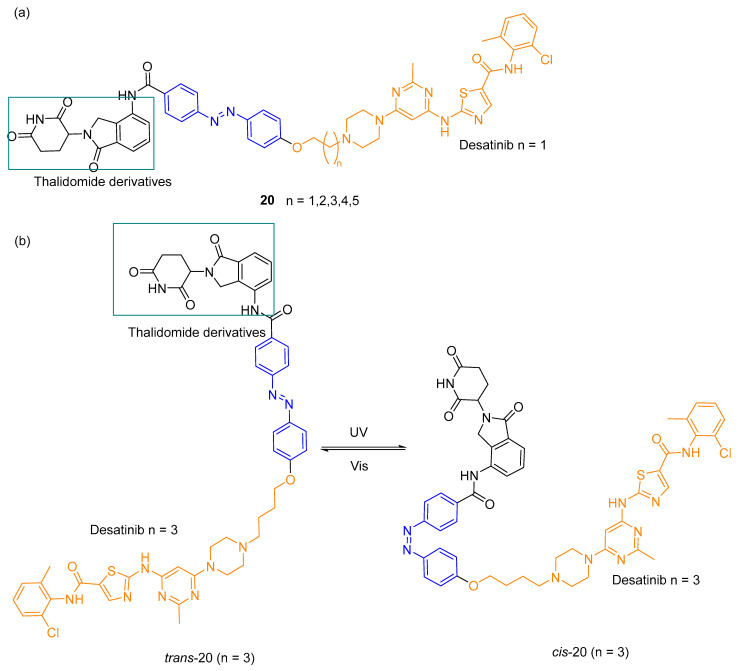
(**a**) Structures of PHOTACs synthesized and (**b**) schematic representation of PHOTAC **20c** (n = 3) and its isomerization [69].

Jin et al. [69] designed PHOTACs (Figure 12a) to knockdown the BCR-ABL fusion protein to interact with E3 ubiquitin ligase cereblon (CRBN) using desatinib, a second-generation tyrosine kinase inhibitor, to target BCR-ABL. The compounds were assessed in a myelogenous leukaemia cell line K-562 and compound **20c** proved to be the most active in reducing BCR-ABL levels selectively by UV irradiation (Figure 12b). The *cis*-isomer did not induce protein degradation.

In general, contrasting with the examples of antibacterial compounds that incorporate azobenzenes (compounds **1**–**2** and **7**–**11**), light does not have an activating effect in some of them. However, in the case of PHOTACs, light triggers a protein destruction reaction. Nevertheless, it is still possible to reverse the process if it is desired to stop such a reaction. Although this is a promising strategy, it is still preliminary and has only been tested in cancer cells.

## 3. Different Classes of Photocleavage and Some Examples in Antibacterial Applications

**Figure 13:**
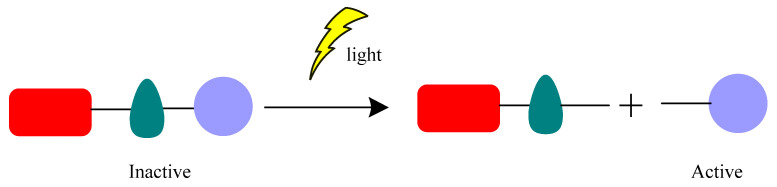
Representation of photocleavage control. Adapted from Friedman [72].

Photocleavage control is another category classified as light-controlled drug activity [72]. In photocleavage, the irradiation triggers the cleavage of a covalent bond between the drug and a light-responsive moiety that is responsible for modulating the drug’s activity (Figure 13).

**Figure 14:**
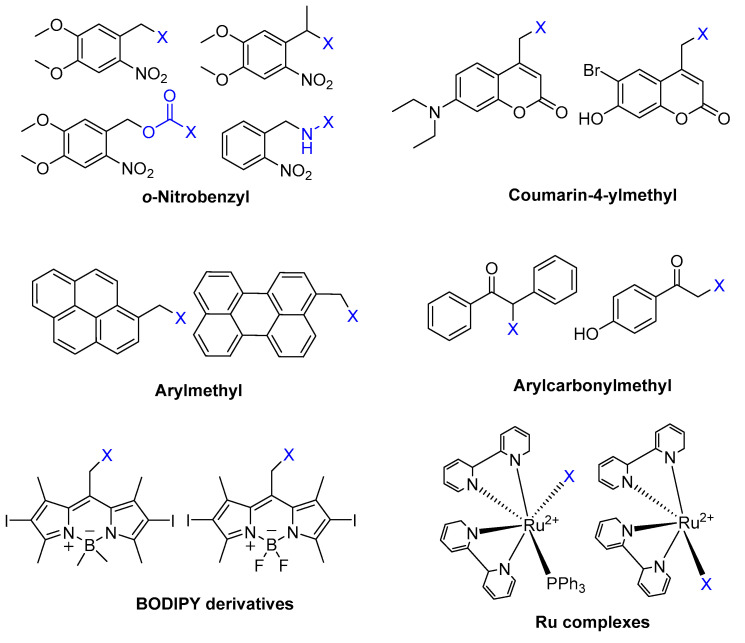
Different types of PPGs. X unit means leaving groups released by photocleavage [7].

Photocleavage reactions need photoremovable protecting groups (PPG). PPG are small light-responsive units that can be covalently linked to the bioactive molecule. By light irradiation, PPG releases, irreversibly, the molecule in its active form [7]. Six classes of PPGs have already been investigated, including *o*-nitrobenzyl groups, coumarin-4-ylmethyl groups, arylmethyl groups, arylcarbonylmethyl groups, boron-dipyrromethene (BODIPY), and Ru complexes (Figure 14).

As mentioned above, for light-controlled drug delivery, it is important to achieve photocleavage groups at long-wavelength light (between 650 nm and 900 nm), such as red light or near infra-red light (NIR), for clinical use [73]. Therefore, BODIPY [74] and Ru complexes [74] have attracted research for having long-wavelength light PPGs and their photolysis process occurring under visible or NIR light irradiation. However, BODIPY derivatives have shown cytotoxic effects due to ^1^O_2_ generation, but there are studies in which after irradiation, this cytotoxicity has been applied to exert a synergistic effect combined with chemotherapy drugs [75].

The use of PPGs has the greatest interest in some biomedical applications, such as to treat of skin infections, cancer, neuronal and antibacterial diseases, and other cellular processes [76].

### 3.1. BODIPY 

**Figure 15:**
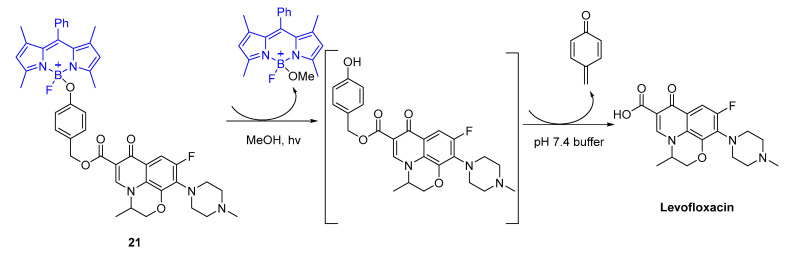
Mechanism proposed for generation of levofloxacin after photoirradiation with blue light (470 nm).

Kumari and co-workers [77] designed a BODIPY complexed with levofloxacin (BODIPY-Levo) to deliver fluoroquinolone upon visible blue light (470 nm) irradiation (Figure 15).

The antimicrobial activity was assessed against *E. coli* and *S. aureus* bacterial strains. Before irradiation, compound **21** did not contribute to the inhibitory effects towards *E. coli* and *S. aureus*. Upon light conditions (470 nm), after releasing levoflaxacin (5.0 µM), the optical density measurement at 600 nm (OD_600_) decreased significantly in comparison to the compound **21** (*p*-value < 0.001), corroborating the rationale of the design [77].

### 3.2. o-Nitrobenzyl Derivatives

Shchelik et al. [78] investigated two ‘’caged’’ antibiotics (vancomycin and cephalosporin, both of which inhibit bacterial cell wall biosynthesis), where the drug was planned to be photoreleased upon UV light exposure (365 nm, 5 min), using *o*-nitrobenzyl as a PPG group and polyethylene glycol (PEG) as a linker (Table 5). PEGylation was an approach to allow steric hindrance, which could suppress vancomycin’s activity and prevent the binding to the amino acid residues of the peptide chain during cell wall synthesis. Cephalosporin PEGylation was expected to prevent binding to penicillin-binding protein or prevent transport issues.

The antibacterial activity was evaluated against *Bacillus subtilis* (*B. subtilis*) ATCC 6633, *S. aureus* ATCC 29,213, MRSA, *E. coli* ATCC 25922, and *P. aeruginosa* ATCC 27853, with MIC values calculated (Table 6).

Compound **22** containing PEG did not show significant activity. However, compound **24** (without PEG) revealed excellent activity against *B. subtilis* and *S. aureus*, as well as vancomycin (compound **23**). In the case of the cephalosporin series, contrary to expectations, PEG decreased the antibiotic activity (compound **25**). Compound **27** exhibited better activity than cephalosporin against all the strains except *P. aeruginosa*, but the activity was revealed to be more efficient in Gram-negative strains, which could be explained by the thiadiazole cephalosporin side chain and its zwitterionic properties that allow higher penetration through the outer membrane (present only on Gram-negative strains). The PEG linker decreased the antibacterial activity against all the tested strains, both in vancomycin and cephalosporin series.

**Figure 16:**
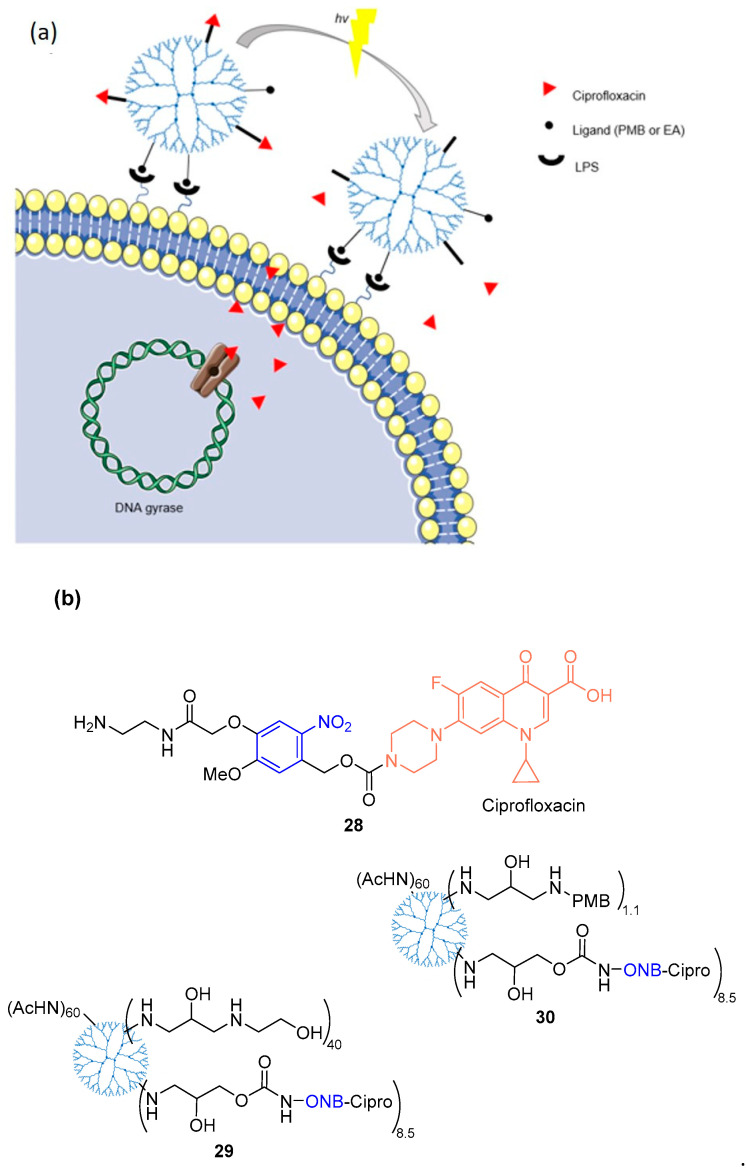
(**a**) Scheme for light-controlled targeted delivery of photocaged ciprofloxacin carried by lipopolysaccharide (LPS) adsorbed to the outer membrane of the Gram-negative bacterium (*E. coli*) cell wall. Internalized ciprofloxacin inhibits DNA gyrase. (**b**) Structures of ciprofloxacin, photocaged ciprofloxacin (**28**), dendrimers conjugated with ONB-ciprofloxacin and cell wall targeting ligand—EA (**29**), or PMB (**30**). Adapted with permission from [79]. Copyright, 1996, Royal Society of Chemistry.

Wong and co-workers [79] reported a delivery mechanism targeted to Gram-negative bacteria based on ciprofloxacin photoreleased by a cell wall-targeted dendrimer nanoconjugate (Figure 16a). The delivery system is a dendrimer conjugated with polymyxin B (PMB) or ethanolamine (EA) that acts as a carrier for ciprofloxacin (inhibitor of DNA gyrase), using the *o*-nitrobenzyl as PPG (ONB-cipro). When PMB or EA binds to lipopolysaccharide (LPS), 80% of ciprofloxacin is photoreleased from ONB-cipro by irradiating UV-A light (365 nm) for 30 min. The antibacterial activity of the conjugates (**29** and **30**, Figure 16b) was evaluated and MIC_50_ values were 2.0 µM (in *E. coli*) approximately, which may be due to an incomplete drug release. The MIC_50_ value of ciprofloxacin is 0.0125 µM. However, the molecules did not show phototoxicity in human cells and exhibited good selectivity for *E. coli*.

### 3.3. Photocaged PROTACs 

**Figure 17:**
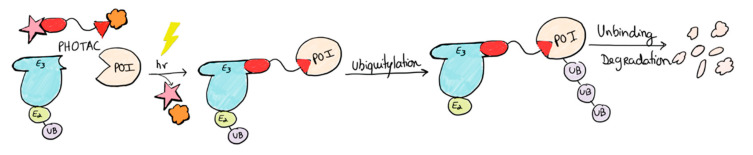
Schematic of the working model of a photocaged PROTAC. The caging moiety attached to either end of the PROTAC is removed upon irradiation. The red geometric figures are in the presence of the PPG groups (pink and orange figures) that prevent the structure from binding to the E3 ubiquitin ligase and POI. Upon irradiation, the PPG groups leave, allowing the structure to bind and, therefore, the ubiquitylation process occurs. Adapted with permission from [80]. Copyright, 2020, John Wiley and Sons.

As in the case of photoswitches, photocaged PROTACs are an approach to photocleavage in which E3 ubiquitin ligase (a linker with a photocleavage moiety) and the POI, form a ternary complex to lead proteasomal degradation [80] (Figure 17). However, photocaged PROTACs are irreversible, unlike photoswitchable PROTACs, which are reversible. Several researchers reported photocaged PROTACs to induce protein degradation by light.

**Figure 18:**
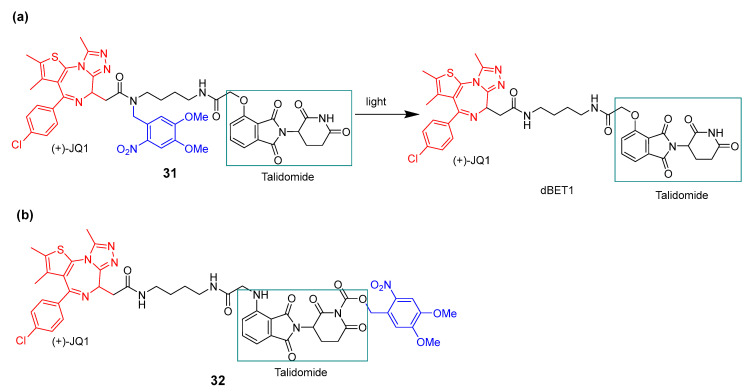
(**a**) Uncaging reaction of photocaged PROTAC **31** in dBET and (**b**) structure of photocaged PROTAC **32**.

Xue et al. [81], for the first time, designed two photocaged PROTACs based on dBET1 PROTAC [71], which used thalidomide as a ligand of E3 ubiquitin ligase cereblon (CRBN) and (+)-JQ1 as a ligand of BRD4. The 4,5-dimethoxy-2-nitrobenzyl (DMNB) introduced is a PPG that is cleaved upon irradiation (365 nm). The photocaged PROTAC **31** (Figure 18a) has the PPG group linked to the amide of (+)-JQ1, and the photocaged PROTAC **32** (Figure 18b) has the PPG group through the imide of thalidomide. Upon exposure to irradiation, photocaged PROTAC **31** generated approximately 50% of dBET1 production, whereas no dBET1 production was observed with photo-caged PROTAC **32**.

Ramos cells were used to assess in vitro BRD4 degradation. BRD4 levels decreased when photocaged PROTAC **31** was irradiated (365 nm, 3 min), being almost completely degraded after 4 h. A zebrafish model was used to assess the in vivo activity of photocaged PROTAC **31,** which showed effectiveness.

**Figure 19:**
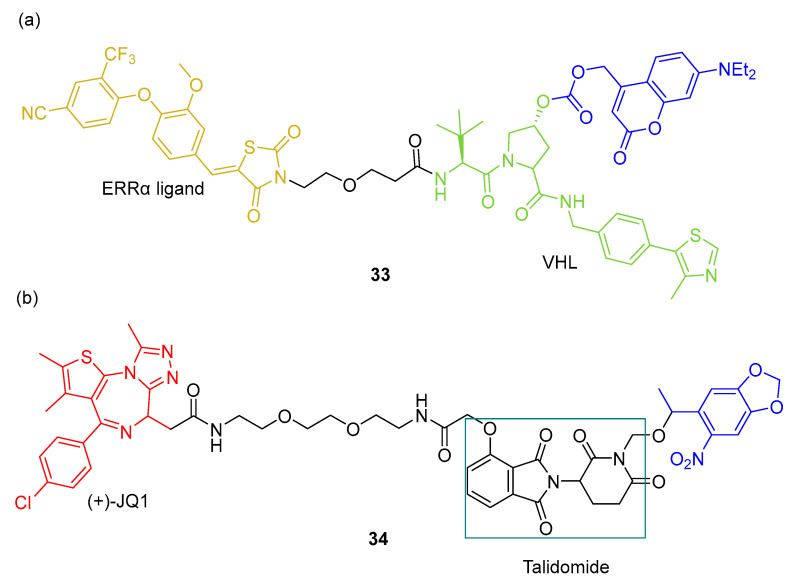
Chemical structures of (**a**) photocaged PROTAC **33** and (**b**) photocaged PROTAC **34**. Upon irradiation, PPG groups are released [82].

Naro et al. [82] also designed other two different photocaged PROTACs using two different PPG groups and ligands recruiting E3 ubiquitin ligases (VHL and CRBN). Photocaged PROTAC **33** (Figure 19a) contains a VHL ligand, targets ERRα (estrogen related receptor α), and uses a diethylaminocoumarin as a PPG group. Photocaged PROTAC **34** (Figure 19b) has a CRBN ligand and targets BRD4 using a 6-nitropiperonyloxymethyl as a PPG group.

MCF-7 cells were used to test the ability of the PPG group of the photocaged PROTAC **33** to block ERRα degradation, and it was verified that the degradation of ERRα was blocked before irradiation. In the case of photocaged PROTAC **34,** HEK293T cells were used to test the degradation of BRD4. In the absence of light, no degradation was verified. Upon exposure to UV-A (365 nm) light, BRD4 was degraded, corroborating the expected results of the designed study. 

**Figure 20:**
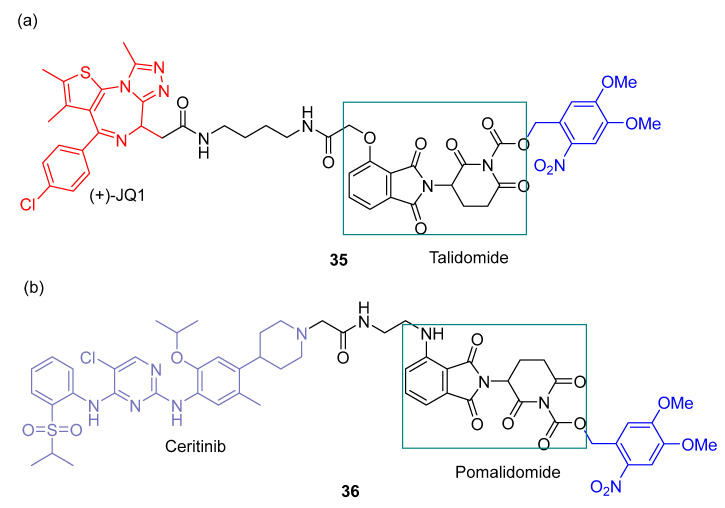
Chemical structures of photocaged PROTAC 35 (**a**) and photocaged PROTAC 36 (**b**).

Jing et al. [67] added a PPG group on pomalidomide based on dBET and dALK PROTACs—photocaged PROTAC **35** (Figure 20a) and photocaged PROTAC **36** (Figure 20b). Dimethoxy-2-nitrobenzyl was used as a PPG group that will prevent pomalidomide from binding to CRBN E3 ubiquitin ligase. In this way, protein degradation will not occur. This will only occur when there is UV-A irradiation, in which PPG leaves and thus pomalidomide can already recruit CRBN ligase for degradation to occur.

HEK293FT cells were used to test the effect of photocaged PROTAC **35** to degrade BRD3-4 by UV-A irradiation. SU-DHL-1 cells were used to assess the effect of opto-dALK to degrade anaplastic lymphoma kinase (ALK, a fusion protein) by UV-A irradiation. Upon UV-A irradiation, photocaged PROTAC **35** is uncaged and leads to the induction of BRD3-4 degradation. On the other hand, when photocaged PROTAC **36** is irradiated with UV-A light, it is also uncaged and promotes the degradation of ALK.

**Figure 21:**
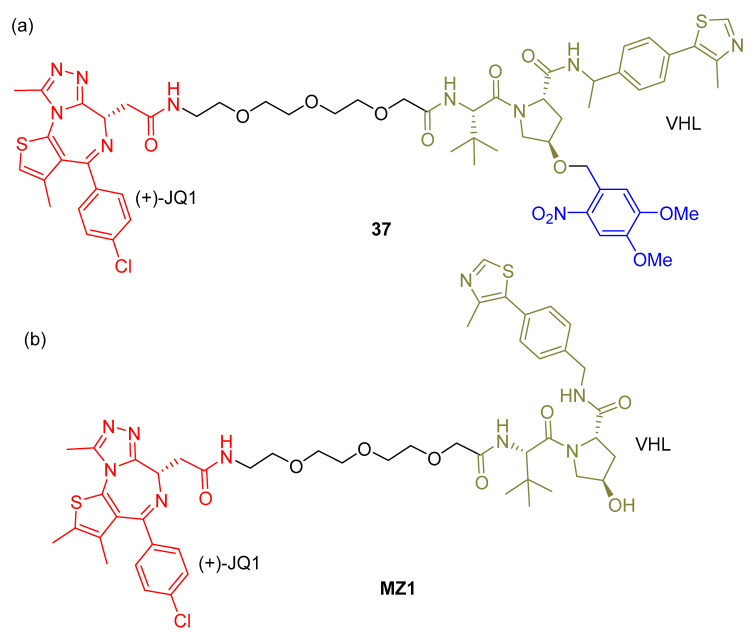
Chemical structures of photocaged PROTAC **37** (**a**) and PROTAC MZ1 (**b**).

Kounde and co-workers [83] designed a photocaged PROTAC (Figure 21a) using a DMNB group binding to VHL E3 ligase-recruiter ligand and (+)-JQ1 as an inhibitor of BET. This approach was based on PROTAC MZ1 [84] (Figure 21b). The DMNB moiety was used to block the recruitment of VHL E3 ubiquitin ligase without irradiation, but it was removed upon UV-A light (365 nm) irradiation. 

The ability of photocaged PROTAC **37** to degrade BRD4 was tested in HeLa cells and its knockdown in real time was performed using live-cell fluorescence imaging (HEK293 cells). The results of this study proved to be good as BRD4 degradation was achieved.

## 4. Porphyrins Combining Photoswitch Systems and Some Application Examples

**Figure 22:**
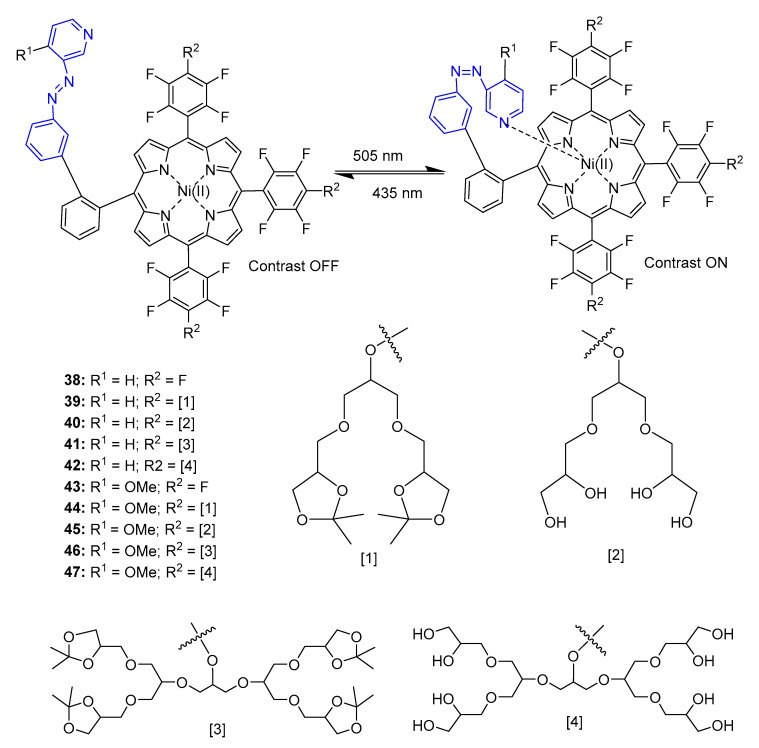
Structures of porphyrins irradiated with 505 nm (contrast ON) and reversible process (435 nm, contrast OFF) [85].

Usually, gadolinium complexes are applied to improve magnetic resonance imaging (MRI) structural contrast in tissues. Light can be used as a functional MRI contrast with high-spatiotemporal resolution. A light-responsive contrast agent for magnetic resonance imaging based on a Ni(II) porphyrin derivative and a switch moiety has previously been reported in the literature [85]. This agent can be applied non-invasively to switch magnetic resonance imaging contrast ON and OFF with a high-spatiotemporal resolution by light stimulus (Figure 22).

Porphyrin derivative **40** was found not to be soluble in water due to the first generation of small glycerol dendrimers, so the hydrophobic character of the Ni(II) porphyrin prevails. However, compound **42** (second generation glycerol dendrimers) prevents porphyrin aggregation and thus increases water solubility. Non-dendronized porphyrins (compounds **38** and **43**) form dimers and with increasing concentration become paramagnetic, decreasing the efficacy of contrast switching. Second generation dendrimers prevent this phenomenon. 

The *trans*-isomer was found not to coordinate intramolecularly, being diamagnetic and thus is MRI silent (contrast OFF), whereas since the *cis*-isomer coordinates intramolecularly, it is paramagnetic and MRI active (contrast ON). Substitutions on R^1^ with pyridine (R^1^ = H) furnished porphyrins with a PSS of 65% *cis*-isomer, while 4-methoxyl (R^1^ = OMe) showed a PSS > 95% *cis*-isomer, allowing the retention of diamagnetic to paramagnetic switching. It was concluded that compound **47** provided the best structures in this study.

**Figure 23:**
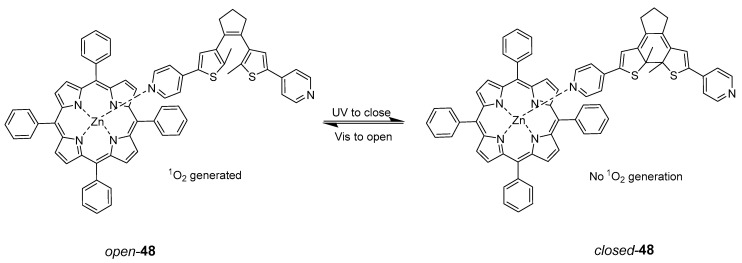
Singlet-oxygen control upon opening and closing photoswitch. The open form (open-**48**) generates ^1^O_2_, while the closed is not active in producing ^1^O_2_.

In the literature, photoswitchable micelles were applied for the control of singlet oxygen (^1^O_2_) generation in PDT, using a zinc(II) complex of 5,10,15,20-tetraphenylporphyrin (ZnTPP) as a photosensitizer and 1,2-bis(5-(4-carbonxyphenyl)-2-methylthien-3-yl)cyclopent-1-ene (BDTE) as a photoswitch (compound **48**, Figure 23). Compound **48** was encapsulated in micelles to cause tumour and bacterial cell death or prevent cell damage upon irradiation through ^1^O_2_ generation [86]. The photoswitching was evaluated in a subcutaneous tissue model.

## 5. Porphyrins Combining Photocleavage Systems and Some Application Examples

**Figure 24:**
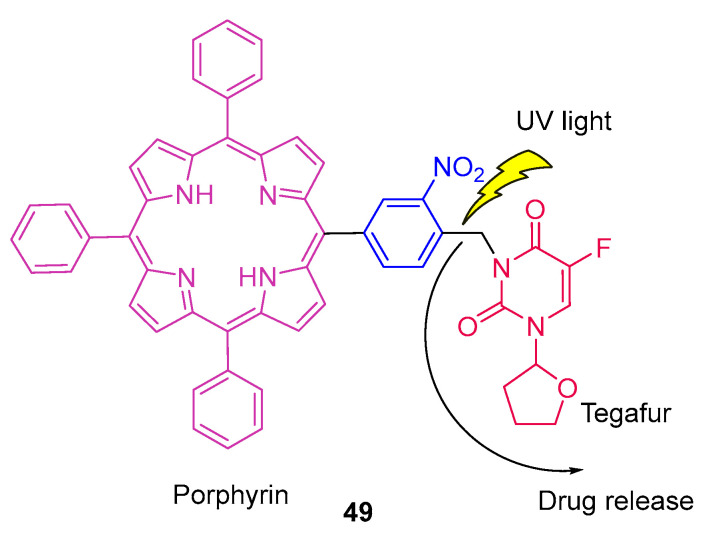
Schematic representation of compound **49** (anticancer prodrug composed by a porphyrin, a PPG, and a parent anticancer drug, tegafur) and its release upon UV-A irradiation [87].

Lin et al. [87] reported for the first time in 2008, an anticancer prodrug (compound **49**, Figure 24) based on an *o*-nitrobenzyl group coupled with a porphyrin. The porphyrin anticancer prodrug is composed of three parts, such as a porphyrin, a PPG (*o*-nitrobenzyl group), and a parent anticancer drug (Tegafur). First, the drug is released by irradiation with UV-A light (350 nm). After 12 min, it achieved a 50% conversion of compound **49** into tegafur. 5-Fluorouracil as an anticancer drug was used as a control, and it was released in the same way as Tegafur.

The authors used MCF-7 human breast adenocarcinoma cells to evaluate the cytotoxicity of compound **49** in the absence and presence of irradiation. In the absence of UV-A light, tegafur induced 91% of cell death while the compound induced only 7% of cell death (less cytotoxic). When this entity is irradiated and, therefore, Tegafur is released, 69% of cell death is observed. Although there is less cell death, this approach allows for minimizing the side effects due to the tumour-affinity property of porphyrin and the light-controllable system.

**Figure 25:**
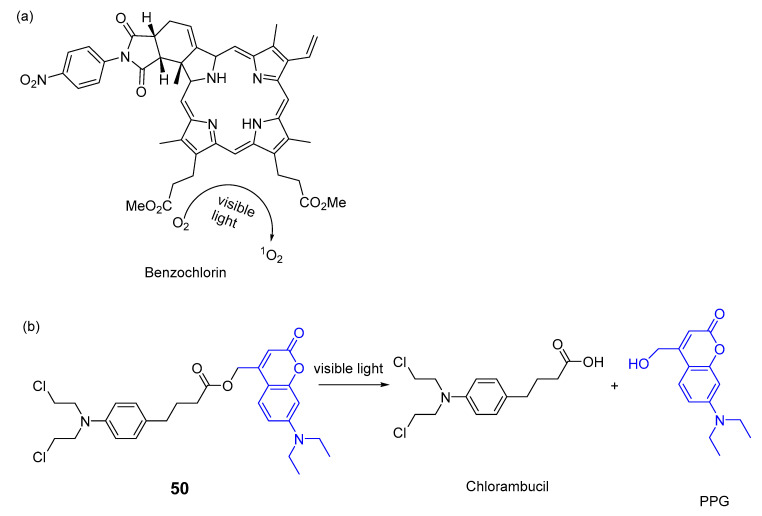
Benzochlorin used as a PS for PDT (**a**) and coumarin-photocaged (compound **50**) release chlorambucil (**b**) [88].

Tessaro et al. [88] reported a novel photocontrolled nanoplatform comprising two photoresponsive components—a benzochlorin PS (Figure 25a) for PDT and a coumarin-photocaged chlorambucil (compound **50**, Figure 25b)—into core-shell micelles to amplify the anticancer activity against MCF-7 human breast adenocarcinoma cells. The authors demonstrated that with simultaneous irradiation with visible light in the micelles, benzochlorin produces ^1^O_2_ as a PS and compound **50** acts as a prodrug by leaving of PPG group (Figure 25b).

Results showed chlorambucil used alone induces 25% cell death, while micelles containing compound **50** and the sensitizer benzochlorin induce 75% cell death after 20 min of blue irradiation (400 nm). This promising multimodal therapy destroys the tumour in two ways—generating ^1^O_2_ and releasing chlorambucil during the irradiation procedure, showing a synergistic effect.

## 6. Porphyrins with Antibacterial Activity

Antimicrobial photodynamic treatment (aPDT) requires, besides an adequate PS, a sufficient PS concentration and an adequate light dose to guarantee full microbial inactivation. However, achieving these conditions is not always easy. To overcome this problem, an ongoing approach that has been presented by the scientific community is the combination of PDT and chemotherapy, as such high concentrations of PS are not necessary because the antibiotic is also used in combination. Thus, the synergistic effect between aPDT and antibiotic therapy is a promising approach as it is possible to increase aPDT efficiency at concentrations below the MIC [89,90]. This section will be presented as an overview of using porphyrins as PS for aPDT and antibiotic therapy combination.

**Figure 26:**
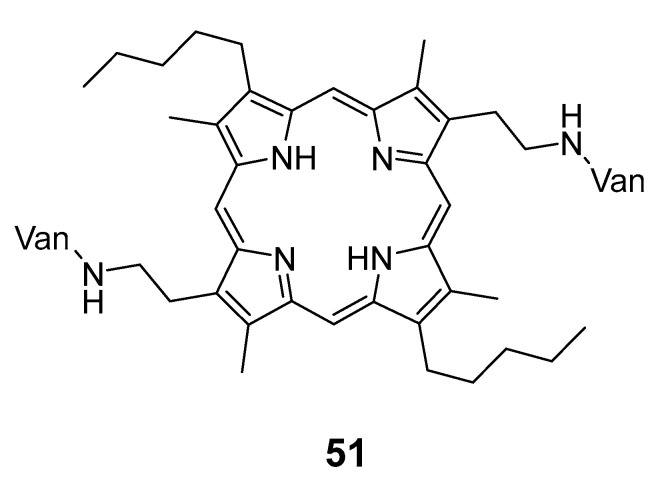
Chemical structure of the conjugated Van-porphyrin. Van = vancomycin [91].

Xing et al. [91] conjugated vancomycin, a glycopeptide antibiotic, with a porphyrin derivative to generate a divalent dimeric system (Figure 26), which has a rigid structure and provides a steric hindrance necessary for interactions between vancomycin and the bacterial strain. The antibacterial activity of compound **51** was tested in *B. subtilis* (ATCC 33,677), *Enterococcus faecium* (*E. faecium)* ATCC 51559, and *Enterococcus faecalis (E. faecalis)* ATCC 51,299, vancomycin-resistant enterococci strains. The results showed a strong photoinactivation activity for the conjugate when compared to vancomycin and porphyrin alone.

**Figure 27:**
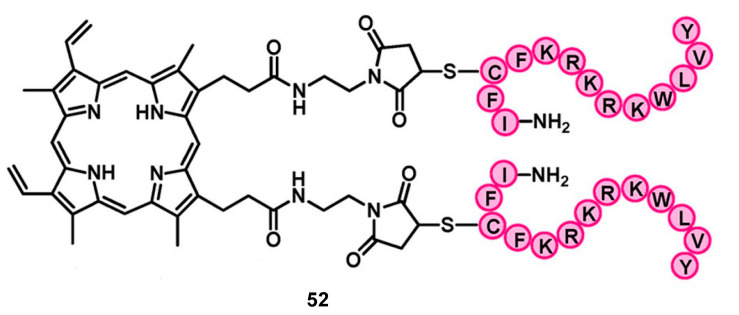
Dimeric PpIX−peptide conjugate. Reprinted with permission from [92]. Copyright, 2012, American Chemical Society.

Liu et al. [92] also developed an approach based on the bioconjugation of a protoporphyrin IX (PpIX) as a PS with lipopolysaccharide (LPS) and an antibacterial peptide (YI13WF) to photoinactivate Gram-negative bacterial strains (Figure 27).

The MIC values (Table 7) were calculated in *E. coli DH5a* (ATCC 53868), *S. enterica* (ATCC 14028), *E. coli* BL21, and *K. pneumoniae* (ATCC 700603). The antibacterial peptide sequence (YI13WF) binds strongly towards lipids at the outer membrane of Gram-negative bacteria, showing potent MIC values against the strains. However, the MIC values of the dimeric conjugate (compound **52**) demonstrated greater aPDT. The antibacterial activity of PpIX alone as a control was also evaluated. The results of the conjugates proved to be significantly better than these components alone.

**Figure 28:**
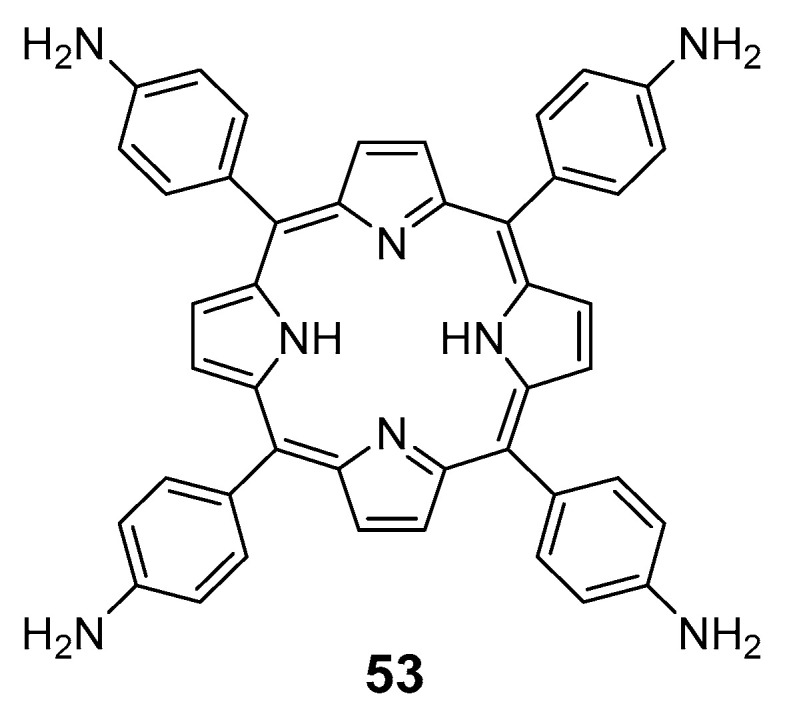
Chemical structure of 5,10,15,20-tetrakis(4-aminophenyl)porphyrin (TAPP).

Dastgheyb and co-workers [93] tested a 5,10,15,20-tetrakis(4-aminophenyl)porphyrin (TAPP) combined with antibiotics used for the treatment of bacterial infections, assuming that combining TAPP with antibiotics would have a complementary effect (Figure 28). TAPP specifically targets bacterial membranes, while tobramycin and chloramphenicol inhibit protein synthesis, and ceftriaxone and vancomycin impair membrane integrity. Results indicated that TAPP with chloramphenicol or tobramycin had an additive effect on *S. aureus* and *E. coli* and a synergic effect on MRSA and *S. epidermidis*. On the other hand, vancomycin and ceftriaxone show only modest effects when combined with TAPP.

**Figure 29:**
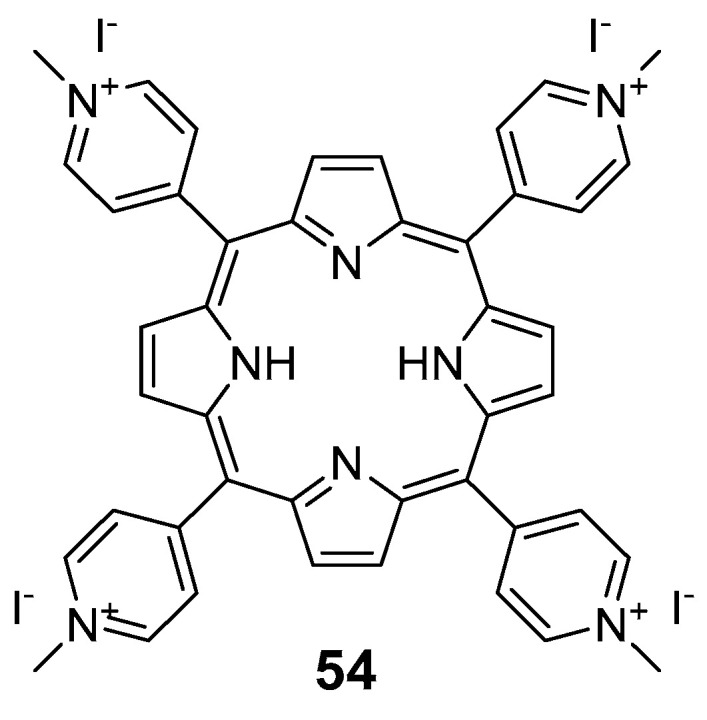
Chemical structure of the cationic porphyrin **54**, also known as TMPyP or Tetra-Py^+^-Me.

Almeida et al. [94] aimed to allow the efficient treatment of aPDT on multidrug-resistant bacteria in hospital wastewaters. Therefore, a 5,10,15,20-tetrakis(1-methylpyridinium-4-yl)porphyrin tetra-iodide **54** (TMPyP, Figure 29) as the PS was used in combination with chloramphenicol. The antibacterial activity of the conjugate was performed in *E. coli* and it was proved that the combination effect of the conjugates was more efficient than TMPyP used alone. The synergic effect could be due to the aPDT cell membrane and the cell wall destabilization, allowing an easier entry of chloramphenicol. 

Branco and co-workers [90] compared the effect of aPDT and aPDT combined with antibiotics to treat skin infected with *S. aureus* ATCC 6538. As well as in the previous work, TMPyP (compound **54**, Figure 29) was used as the PS. The antibacterial activity was performed in vitro and ex vivo (porcine skin), using compound **54** combined with and without antibiotics. In vitro, a bacterial reduction of ~8 log after 180 min of white light irradiation at an irradiance of 4.0 mW cm^−2^ was attained using 5.0 μM of **54** without antibiotic. When tested, the combined action of 0.5 and 1.0 μg mL^−1^ ampicillin with 5.0 μM of **54** increased the efficacy, observing a reduction of ~8 log after 60 min and 30 min of white light irradiation, respectively. When the ampicillin was tested alone, no colony-forming unit (CFU) reduction was observed, even at the highest tested concentration, 1.0 μg mL^−1^. In ex vivo experiments, using 50 μM of **54** after 180 min of irradiation with white light at an irradiance of 150 W m^−2^, a decrease of ~4 log (99.99% reduction) of bacterial abundance was observed. However, when treated the porcine skin simultaneously with TMPyP (50 μM) and with 5.0 μg mL^−1^ ampicillin, a bacterial reduction of ~5.6 log (>99.9995% reduction) after 180 min of white light irradiation was observed. Using the antibiotic alone at this concentration, only a CFU reduction of ~1 log was noted.

**Figure 30:**
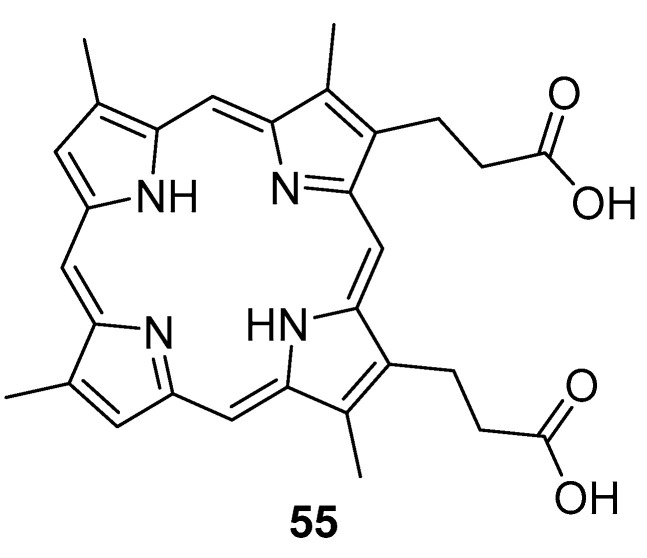
Chemical structure of the deuteroporphyrin **55** (DP).

Iluz et al. [95] searched for a synergistic effect between deuteroporphyrin **55** (Figure 30) and antibiotics usually used to treat infections with *S. aureus* ATCC 25,923 and MRSA. Although gentamicin, vancomycin, rifampin, and fusidic acid treatments were tested in combination with aPDT, only oxacillin (1 μg mL^−1^) proved to have a synergistic effect in combination with deuteroporphyrin (4 μM), during 24 h, at a light dose of 15 J·cm^−2^, showing a CFU reduction of ~5.5 log (99.9995% reduction). Cultures treated with monotherapy (oxacillin or **55** alone) showed no log decrease (0% reduction), even though there was an increase during 2h of treatment. 

## 7. Concluding Remarks and Future Challenges

The problem of antimicrobial and chemotherapeutic resistance is that is results frequently in therapeutic failure. With the emergence of new strains of resistant bacteria, the search for effective antibiotics has become a critical global health priority and has motivated several research groups around the world to find effective alternatives.

Photodynamic treatments, photoswitches, photocaged groups, and recent PHOTACs and photocaged PROTACs strategies have been explored as promising approaches to tackle the AMR issue. As it was demonstrated (compounds **3**–**6** and **12**–**15**), photoswitches have the advantage of being reversible compared to photocaged compounds. However, the design of these molecules is not always easy or straightforward because they were related to a series of azobenzene-based compounds where light would not have the activating effect but rather result in deactivation, which is very important for deactivation processes. Despite this, these deactivation approaches will be more relevant in the case of PHOTACs to stop the degradation protein process if necessary. However, many approaches reported using UV-A-C irradiation, which is harmful to healthy cells, or with visible blue light, which has limited tissue penetration. As far as we know, only one example was found in the literature that reports the use of NIR light in azobenzene-based compounds (compounds **5** and **6**). Therefore, it is necessary to improve strategies of incorporation of red-shifted chromophores to address the tissue penetration problem, even in PHOTACs (not just restricted to photoswitchable compounds). Apparently, there are not as many studies done on photocaged compounds as there are on photoswitchable compounds, which could be due to the fact that the process is not reversible. Photocaged PROTACs do not seem to be as promising because of they do not allow protein degradation to be stopped if necessary. Nevertheless, PHOTACs and photocaged PROTACs are reported for cancer treatment, but it should be important to prove their effectiveness for other diseases, such as antimicrobial infections, to combat resistant strains. Furthermore, porphyrins and their derivatives (e.g., chlorins) have been demonstrating a synergistic effect when used with antibiotic therapy. However, the most recent study on this topic was published several years ago, so it is critical to continue researching in this field, specially involving porphyrin derivatives with the antimicrobial activity in aPDT, since no resistance mechanism seems to be developed by the biological entities. It is important to note that, to date, examples of porphyrin derivatives combining photoswitchable or photocaged systems with antimicrobial activity are not described in the literature and there is still room to develop interesting photoswitchable or photocaged systems.

Photoswitches and photocleavage compounds have shown promising potential for use in various clinical applications. However, as with any new therapeutic agent or its combined action, there are potential safety concerns associated with their use that must be evaluated [96]. One of the main safety concerns is the potential risk for these compounds to elicit adverse effects or toxicity. To mitigate these potential safety risks in a clinical context, it is crucial to conduct comprehensive preclinical studies to evaluate toxicity and to find the optimal therapeutical conditions in order to carefully control and avoid toxicity. Additionally, the delivery method must be optimized to ensure that the desired target compounds are only delivered to the target tissues or organs. [97]. Overall, the successful translation of externally activated drug delivery systems from preclinical studies to human clinical trials requires a comprehensive understanding of the complex interactions between the delivery system and the target biological environment [98]. To address these challenges, it is important to employ animal models, assess drug dose and administration routes based on in vitro experiments, and develop effective methods for monitoring the long-term toxicity and pharmacokinetics of the delivery system in vivo [99]. While external triggers pose additional challenges, the imaging capabilities of many triggered delivery systems offer unique opportunities for better monitoring the fate of drugs and drug carriers in vivo [100].

Our aim for this review is to offer new perspectives on the use of photopharmacology, not only for bacterial inactivation to mitigate antibiotic resistance but also for cancer treatment, motivating the medical and scientific communities to devote greater attention and resources to these issues. This, in turn, will enhance the comprehension of the problem, enabling the identification of potential solutions that could lead to improved public health outcomes.

## Figures and Tables

**Table 1:** Minimum inhibitory concentration (MIC) values of ciprofloxacin and their derivatives.

Compound	MIC (μM)			
	Before Irradiation Compound 1		After Irradiation Compound 2	
	*E. coli*	*M. luteus*	*E. coli*	*M. luteus*
Azofloxacin	0.50	0.250	0.50	0.50
Ciprofloxacin	0.0125	12.0	0.0125	12.0

**Table 2:** IC_50_ values for *trans* and *cis* azobenzenes against bacterial HDACs.

IC_50_ (μM)					
Compound	R	B/A-HDAH	PA-HDAH	APAH PA0321	APAH PA1409
**7a (** *trans* **)**	H	0.042	0.0056	0.79	2.0
**8a (** *cis* **)**	H	0.062	0.0066	0.40	0.21
**7b (** *trans* **)**	*t*-But	0.37	0.072	2.2	>25
**8b (** *cis* **)**	*t*-But	0.38	0.094	1.8	3.5
**7c (** *trans* **)**	OMe	0.059	0.051	2.3	5.1
**8c (** *cis* **)**	OMe	0.075	0.090	1.4	0.65
**Vorinostat**		0.095	0.037	0.50	0.30

**Table 3:** MIC values of Gramicidin S and its mimetics peptides against *S. aureus* ATCC 49775.

	MIC (µg mL^−1^)	
Compound	*cis*	*trans*
**9**	64	64
**10a**	64	256
**10b**	32	128
**10c**	>256	>256
**11a**	256	>256
**11b**	>256	>256
**Gramicidin S**	2	

**Table 4:** MIC values of diarylethene-based switchable antibacterial agents before and after UV-C (254 nm) light irradiation.

Compound	MIC (μg mL^−1^)			
	Open-Isomer (before)		Closed-Isomer (after)	
	*E. coli*	*S. aureus*	*E. coli*	*S. aureus*
**12a**	32	8	16	8
**12b**	32	16	2	16
**13a**	16	32	16	32
**13b**	16	32	16	32
**14a**	32	16	8	16
**14b**	32	16	2	16
**15a**	32	16	16	16
**15b**	16	16	4	16
Norfloxacin	0.125	0.125	0.125	0.125
Ciprofloxacin	0.125	0.125	0.125	0.125

**Table 5:** Vancomycin (**22**,**23**, and **24**) and cephalosporin (**26**, **27**, and **28**) derivatives.

	Vancomycin Derivatives	Cephalosporin Derivatives
Target Compounds	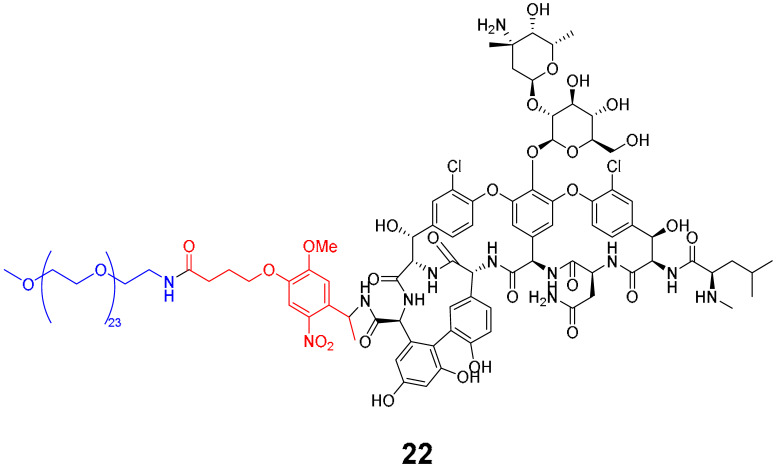	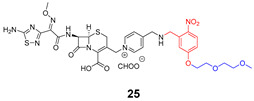
Release Compounds	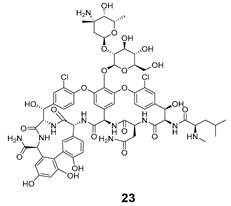	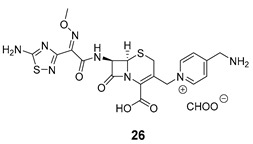
Control Compounds	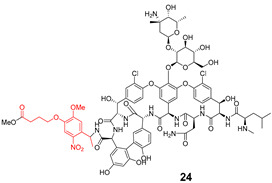	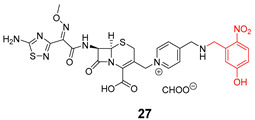

**Table 6:** MIC values (µg mL^−1^) of vancomycin and cephalosporin derivatives.

Strains	MIC (µg mL^−1^)					
	Vancomycin Series			Cephalosporin Series		
	22	23	24	25	26	27
*E. coli* ATCC 25922	-	-	-	8	1–2	1
*P. aeruginosa* ATCC 27853	-	-	-	64	2–4	32
*B. subtilis* ATCC 6633	32	0.06–0.125	0.125	8	2–4	1
*S. aureus* ATCC 6633	>64	0.5–1	0.5	32	8	4
*S. aureus* ATCC 43300	>64	1–2	1	64	32	16

**Table 7:** MIC values, expressed in μM, of the conjugate free peptide YI13WF and PpIX.

	MIC Values (μM)			
	*E. coli* DH5a	*S. enterica*	*E. coli* BL21	*K. pneumoniae*
PpIX−Peptide Conjugate	2.0	4.0	4.0	8.0
YI13WF	8	~24	32	32
PpIX	>64	>64	>64	>64

## Data Availability

Data sharing not applicable.
